# Postpartum Mastitis and Community-acquired Methicillin-resistant *Staphylococcus aureus*

**DOI:** 10.3201/eid1302.060989

**Published:** 2007-02

**Authors:** Pavani Reddy, Chao Qi, Teresa Zembower, Gary A. Noskin, Maureen Bolon

**Affiliations:** * Northwestern University Feinberg School of Medicine, Chicago, Illinois, USA

**Keywords:** Methicillin-resistant, *Staphylococcus aureus*, postpartum mastitis, community-acquired, dispatch

## Abstract

This single-center, case-control study documents a relative increase in methicillin resistance among 48 cases of *Staphylococcus aureus*–associated postpartum mastitis during 1998–2005. Of 21 cases with methicillin resistance, 17 (81%) occurred in 2005. Twenty (95%) isolates contained the *Staphylococcus* cassette chromosome *mec* type IV gene; this suggests that the increase is due to community-acquired methicillin-resistant *Staphylococcus aureus*.

Postpartum mastitis (PPM) occurs in as many as one third of breastfeeding women in the United States and leads to breast abscess formation in ≈10% of cases (*1*,*2*). Although breast milk cultures are not routine in PPM management, the growth of potentially pathogenic bacteria (such as β-hemolytic streptococci or *Staphylococcus aureus*) is associated with longer time to recovery and more frequent abscess formation (*3*). *S. aureus* is the most common bacterium isolated from such cultures, representing 37%–50% of isolates (*4*,*5*).

Reports of methicillin-resistant *S. aureus* (MRSA) PPM among young, healthy women lacking traditional risk factors for MRSA have emerged in the past few years (*6*,*7*). Isolates in these cases of community-acquired infection (CA-MRSA) remain susceptible to multiple non–β-lactam antibiotics and possess distinct molecular features (*8*).

Although risk factors associated with skin and soft tissue infections due to CA-MRSA have been described (*8*,*9*), characteristics unique to patients with CA-MRSA PPM are unknown. To identify risk factors, complications, and outcomes among patients with CA-MRSA PPM, we conducted a retrospective, case-control study to include all *S. aureus*–associated cases at a single institution over an 8-year period. MRSA isolates were analyzed by PCR for the presence of the *Staphylococcus* cassette chromosome (SCC) *mec* type IV gene, which is commonly associated with community-acquired infection.

## The Study

We considered for analysis all patients from Northwestern University’s Prentice Women’s Hospital and affiliated Lynn Sage Comprehensive Breast Center with wound, fluid, drainage, or breast milk cultures positive for *S. aureus* from January 1998 through December 2005. Case-patients were defined as patients with PPM and a corresponding culture positive for MRSA. Control-patients were defined as patients with PPM and a corresponding culture positive for methicillin-susceptible *S. aureus* (MSSA). Patients who had no evidence of mastitis or who had a history of MRSA were excluded from the study. SCC*mec* types I–V were identified by a PCR-based multiplex assay; rapid bacterial DNA extraction and PCR amplification were performed as described elsewhere (*10*).

**Figure 1:**
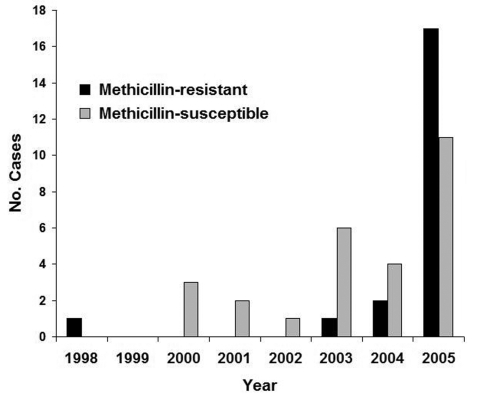
Cases of *Staphylococcus aureus*–associated postpartum mastitis at a single institution, 1998–2005. Cochrane-Armitage test for linear trend suggests a relative increase in methicillin-resistant cases during the study period; p = 0.04.

Forty-eight cases of *S. aureus*–associated PPM were identified during the study period; 21 cases were due to MRSA and 27 cases were due to MSSA. A relative increase in MRSA PPM was noted in the later years of the study (Figure 1, p = 0.04). MRSA and MSSA patients did not differ significantly with respect to age, pregnancy history, or symptoms at the time of initial evaluation. In addition, MRSA and MSSA patients did not differ in terms of potential risk factors for infection, such as diabetes, group B β-hemolytic streptococcus colonization, artificial rupture of membranes, epidural anesthesia, vaginal lacerations, episiotomy, cesarean section, or intrapartum antibiotic use (Table).

**Table:** Demographics, symptoms, interventions, and outcomes in patients with *Staphylococcus aureus*–associated postpartum mastitis*

**Variable**	**No. (%) patients**		**OR (95% CI)**	p value*
	MRSA (n = 21)	MSSA (n = 27)		
Demographics				
Median age, y	32	32.5	–	0.90
Multiparous	12 (57)	9 (33)	2.67 (0.71–10.4)	0.10
Race†				
Caucasian	15 (71)	19 (79)	0.66 (0.13–3.19)	0.55
Other	6 (29)	5 (21)	1.52 (0.31–7.59)	0.55
Clinical symptoms				
Fever	7 (33)	10 (37)	0.85 (0.22–3.30)	0.79
Skin changes or fissures	6 (29)	8 (30)	0.95 (0.23–3.96)	0.94
Induration	20 (95)	21 (78)	5.71 (0.59–275.7)	0.09
Median time from delivery to symptom onset, d	27	33.5	–	0.60
Prenatal risk factors				
Diabetes	2 (10)	1 (4)	2.74 (0.13–167.56)	0.41
Group B β-hemolytic streptococcus colonization‡	3 (16)	4 (16)	0.88 (0.11–5.96)	0.87
Intrapartum risk factors				
Intrapartum treatment with antimicrobial drugs†	9 (43)	7 (29)	1.82 (0.45–7.52)	0.34
Cesarean section‡	3 (14)	2 (8)	1.92 (0.19–24.89)	0.50
Artificial rupture of membranes§	12 (63)	10 (44)	2.23 (0.54–9.41)	0.20
Vaginal laceration or episiotomy§	18 (95)	22 (96)	0.82 (0.01–67.75)	0.89
Epidural anesthesia§	12 (63)	18 (78)	0.48 (0.10–2.26)	0.28
Interventions				
Aspiration	17 (81)	22 (82)	0.97 (0.18–5.66)	0.96
Repeat aspiration	7 (41)	5 (23)	2.38 (0.48–12.14)	0.22
Incision and drainage	1 (6)	9 (41)	0.09 (0.00–0.84)	0.01
Drain placement	3 (14)	6 (22)	0.58 (0.08–3.26)	0.49
Outcomes				
Hospital admission	10	11	1.32 (0.36–4.90)	0.63
Median length of stay, d	4	4	–	0.90
Median leukocyte count, cells/μL	12.8	15.3	–	0.21
Temperature >38.1°C	6 (60)	2 (18)	6.75 (0.69–88.48)	0.05
Recurrent symptoms requiring readmission	1 (10)	1 (9)	1.11 (0.01–95.83)	0.94
Outpatient, later admitted	2 (18)	1(6)	3.75 (0.16–235.66)	0.29
Breastfeeding discontinued¶	3 (16)	5 (22)	0.71 (0.10–4.38)	0.67

*MRSA, methicillin-resistant *Staphylococcus aureus*; MSSA, methicillin-susceptible *S. aureus*; OR, odds ratio for categorical variables using χ^2^ analysis or Fisher exact test where appropriate; CI, confidence interval; p values for continuous variables calculated by using the Wilcoxon test. †Data available for 24 MSSA patients. ‡Data available for 25 MSSA patients. §Data available for 19 MRSA and 23 MSSA patients. ¶Data available for 19 MRSA and 24 MSSA patients.

Ten (48%) MRSA and 11 (41%) MSSA patients required hospitalization. Although these inpatients did not differ in duration of symptoms before admission, length of stay, or leukocyte count, MRSA patients were more likely to have fever. One patient in each group required readmission for recurrent symptoms (Table).

Forty-six study patients had an abscess associated with mastitis; most (39 patients) underwent needle aspiration. Of these patients, 7 (41%) MRSA and 5 (23%) MSSA patients required repeat aspiration. Notably, 9 MSSA patients underwent incision and drainage a median of 4.5 days after aspiration (range 0–17 days), whereas only 1 MRSA patient required subsequent débridement (1 day later). Reasons for this difference are not clear; however, the more frequent use of serial ultrasound-guided aspiration in breast abscess management in recent years (when most MRSA cases occurred) may account for this finding.

In 17 of 21 MRSA cases, antibiotic use was documented. Twelve patients received antibiotics effective against MRSA, but only 2 received effective coverage at therapy onset (both received clindamycin). Patients initially received a penicillinase-resistant penicillin (10 patients), a first-generation cephalosporin (3 patients), a β-lactam/β-lactamase inhibitor (1 patient), or some combination of the above (6 patients). Median time to effective coverage for MRSA was 5 days (range 0–16 days); adequate antimicrobial agents included vancomycin (4 patients), trimethoprim-sulfamethoxazole (1 patient), clindamycin (9 patients), rifampin (2 patients), or some combination of the above (4 patients). Median duration of therapy, documented in 8 of 12 effective regimens, was 19 days (range 14–62 days).

Antimicrobial agent use was documented for 18 of 27 MSSA cases; in all 18 cases, isolates were susceptible to the initial antibiotic of choice. Initial regimens included penicillinase-resistant penicillins (10 patients), first-generation cephalosporins (2 patients), macrolides (1 patients), tetracyclines (1 patients), β-lactam/β-lactamase inhibitors (1 patient), vancomycin (1 patient), and clindamycin (2 patients). Duration of therapy for MSSA PPM, documented in 12 of 18 cases, was a median of 13.5 days (range 9–27 days).

Medical record review of affected patients did not show transmission of *S. aureus* to infants or other family members. In 1 MRSA patient, a perirectal abscess developed 5 months after the mastitis resolved. Intraoperative cultures of the abscess grew MRSA with identical susceptibilities, which suggests persistent colonization; however, typing of the isolates was not performed.

**Figure 2:**
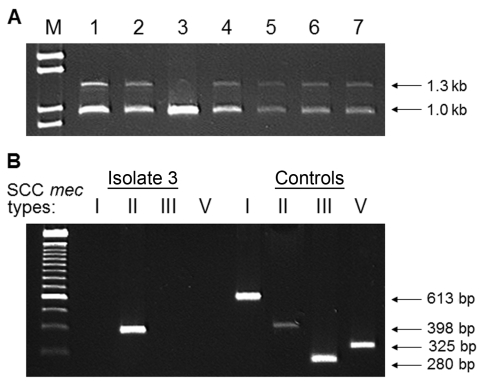
A) PCR with specific primers for class B *mec* complex (1.3 kb) and type 2 *ccr* complex (1.0 kb) identifies isolates containing *Staphylococcus* cassette chromosome (SCC) *mec* type IV: lanes 1, 2, and 4–7. B) When control strains are used, PCR identifies SCC*mec* type II in isolate 3.

Of 21 MRSA isolates available for PCR analysis, 20 possessed SCC *mec* IV. The remaining isolate contained SCC *mec* II (Figure 2) and displayed resistance to clindamycin. In contrast, 95% of isolates with SCC *mec* IV were clindamycin susceptible.

## Conclusions

To our knowledge, this is the largest case-control study of patients with MRSA-associated PPM. Although *S. aureus* is the most common etiologic agent of PPM, cases caused by MRSA have rarely been described. Epidemic MRSA cases, linked to the hospital transmission of a community-acquired isolate, have been observed more recently (*6*). Our study suggests that CA-MRSA is an increasingly common pathogen in spontaneous cases of PPM.

PPM due to CA-MRSA appears to be increasing at our institution. Among 17 MRSA-infected mothers in 2005, delivery dates spanned >9 months without overlap, which suggests that MRSA was independently acquired rather than outbreak-related. In addition, although isolates were not subjected to molecular typing by pulsed-field gel electrophoresis, PCR results suggest that 16 (94%) of MRSA isolates in 2005 were community-acquired.

The epidemiology of CA-MRSA PPM is poorly understood. Notably, nearly twice as many MRSA-infected than MSSA-infected women were multiparous in this study (57% vs. 33%, respectively). The prevalence of CA-MRSA is increasing among young children, and intrafamilial transmission of isolates has been documented (*11*,*12*); therefore, mothers with young children may be at increased risk for CA-MRSA PPM. Alternatively, these patients may serve as a reservoir for MRSA in the community, transmitting this organism to family members.

In the current study, women with MRSA were significantly less likely to receive adequate and timely antimicrobial drug treatment, but consequences of this difference are unclear. Lee et al. suggest that small CA-MRSA abscesses in children can be managed effectively with incision and drainage alone (*13*). Indeed, most women in this study underwent incision and drainage or wound aspiration without significant differences in outcomes. Although MSSA patients were more likely to undergo breast abscess incision and drainage than their MRSA counterparts, both methods are considered appropriate surgical interventions (*14*).

Although related cases of infant infection were not found, charts of household contacts were not reviewed in this study; cases of *S. aureus* transmission to infants or other family members may have been undetected. Several authors have reported mother-to-infant transmission of MRSA through breast milk (*15*,*16*). Although decolonization measures in MRSA-colonized patients have not demonstrated long-term effectiveness (*17*), the possibility of infant MRSA acquisition may warrant further evaluation of such measures in infected, breastfeeding mothers.

As with any retrospective case-control study, ours had several limitations. First, the study population is small, which limits the generalizability of the results. Second, patients were added to the study by using results of positive cultures; consequently, cases likely represented more severe and complicated infections in which cultures were necessary after routine therapeutic measures failed. Third, although PPM has been associated with multiple patient factors (i.e., difficulty breastfeeding, tobacco use, and stress), a thorough risk assessment is limited by retrospective study. In addition, medical record review may not indicate certain CA-MRSA risk factors, such as socioeconomic status, history of incarceration, or exposure to day care facilities. Finally, although the study results suggest a recent increase in MRSA PPM, an assessment of incidence would require further prospective analysis.

In summary, CA-MRSA has emerged as an increasingly common pathogen in PPM. Therapy against CA-MRSA should be considered in refractory or severe cases of PPM until wound, drainage, or breast milk cultures can be obtained. Adjunct surgical drainage or aspiration is often warranted in such cases. Additional study is required to determine the utility of routine cultures in postpartum mastitis, the prevalence of CA-MRSA in this emerging problem, and the consequences of CA-MRSA colonization for breastfeeding infants.
